# Pulse-gated noncontrast thoracic magnetic resonance angiography for acute aortic dissection with transient ischemic attack: A case report

**DOI:** 10.1016/j.ensci.2021.100329

**Published:** 2021-02-20

**Authors:** Takeshi Bo, Yasuhito Kawana, Itsuki Soejima, Eiichiro Amano, Tetsuya Komatsuzaki, Jun Oyama, Akira Machida

**Affiliations:** a Department of Neurology, Tsuchiura Kyodo General Hospital, Japan; b Department of Radiology, Tsuchiura Kyodo General Hospital, Japan; c Department of Diagnostic Radiology and Nuclear Medicine, Tokyo Medical and Dental University, Japan

**Keywords:** Thoracic magnetic resonance angiography, Balanced steady-state free precession imaging, Aortic dissection, Transient ischaemic attack, TIA, transient ischemic attack, CTA, computed tomographic angiography, MRI, magnetic resonance imaging, MRA, magnetic resonance angiography, bSSFP-MRA, balanced steady-state free precession, MRA, TAAAD, type A acute aortic dissection, TOF-MRA, time-of-flight MRA

## Abstract

Aortic dissection is a rare cause of an acute ischemic stroke or transient ischemic attack (TIA). Aortic dissection is particularly challenging in stroke patients who are eligible for thrombolysis secondary to the diagnostic difficulty within a narrow time window (4.5 h) and have a risk of developing life-threatening hemorrhagic complications following thrombolysis. Computed tomographic angiography (CTA) has been the mainstay of imaging when evaluating acute aortic syndrome. However, it cannot be routinely performed for pregnant patients and those with renal failure or iodine-contrast media allergy. We report a case of a 72-year-old woman who developed transient right-hand paralysis without any chest symptoms. Brain magnetic resonance imaging (MRI) showed no recent infarction; however, the brachiocephalic trunk was not well visualized on carotid magnetic resonance angiography (MRA). Subsequent thoracic pulse-gated noncontrast three-dimensional balanced steady-state free precession MRA (bSSFP-MRA) detected a Stanford type A acute aortic dissection (TAAAD). This was confirmed by CTA, leading to the diagnosis of TIA due to Stanford TAAAD. Pulse-gated noncontrast thoracic bSSFP-MRA was acquired a few minutes after a series of brain MRI scans. This imaging modality is expected to be used as a screening platform to rule out Stanford TAAAD during the hyperacute phase of stroke.

## Highlights

• CTA has been the mainstay of imaging when evaluating acute aortic syndrome.
• bSSFP-MRA can detect Stanford type A acute aortic dissection.
• Pulse-gated noncontrast thoracic bSSFP-MRA can screen for Stanford TAAAD.

## 1 Introduction

Painless Stanford type A acute aortic dissection (TAAAD) is rare and seldom causes an acute stroke or transient ischemic attack (TIA) [1,2]. However, screening must be performed to exclude TAAAD even when it is not clinically suspected. Here, we report a case of a 72-year-old woman with TIA secondary to TAAAD, in whom thoracic pulse-gated noncontrast three-dimensional balanced steady-state free precession magnetic resonance angiography (bSSFP-MRA), obtained with a 1.5-T MR scanner (INGENIA), helped diagnose aortic dissection.

## 2 Case report

**Fig. 1:**
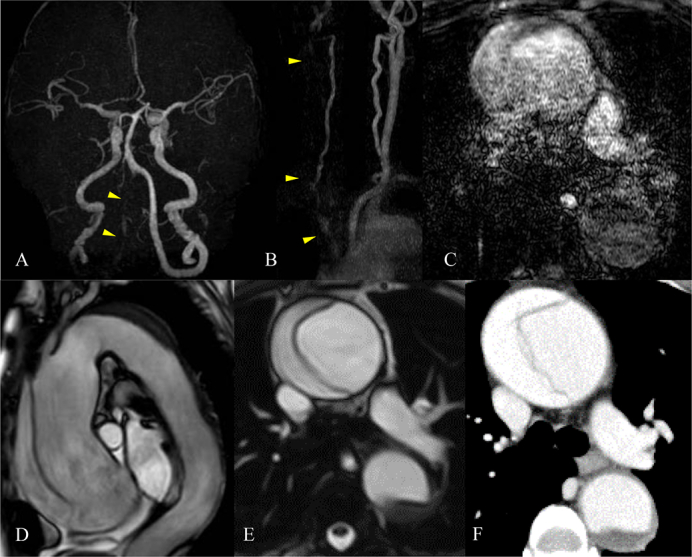
CT and MR imaging of the brain, neck, and thoracic aorta. The right distal VA in the brain MRA (yellow allow) and the right ICA show a slightly lower signal intensity than the left ICA (A). The brachiocephalic artery in the cervical MRA is invisible (yellow arrow), and the right vertebral artery can be partially observed (B). The oblique sagittal pulse-gated noncontrast thoracic bSSFP-MRA image clearly shows a dissection flap in the dilated ascending aorta (D). Comparing TOF-MRA (C), pulse-gated noncontrast thoracic bSSFP-MRA (E), and chest CTA (F), pulse-gated noncontrast thoracic bSSFP-MRA produced a better imaging quality for flap detection. CT, computed tomography; MR, magnetic resonance; VA, vertebral artery; ICA, internal carotid artery; MRA, magnetic resonance angiography; bSSFP, balanced steady-state free precession; TOF, time-of-flight; CTA, computed tomographic angiography. (For interpretation of the references to colour in this figure legend, the reader is referred to the web version of this article.)

A 72-year-old woman presented 3 h after a sudden onset of a right arm palsy. Her only stroke risk factor was hyperlipidemia. She reported no chest or back pain, and her arm paralysis had improved prior to the presentation. Neurologic examination revealed no symptoms on arrival; the National Institutes of Health Stroke Scale score was 0. The left arm blood pressure was 128/49 mmHg, and heart rate was 64 beats per minute. The right arm blood pressure was not checked because aortic dissection was not suspected. She was fully awake (Glasgow Coma Scale score = 15). She had normal heart sounds. The pretibial pitting edema and jugular venous distension were not observed. Laboratory tests revealed elevated D-dimer (50.5 μg/mL, normal <0.5 μg/mL). Electrocardiography showed normal sinus rhythm. Brain magnetic resonance imaging (MRI) showed no areas of restricted diffusion consistent with acute cerebral infarction. The distal right vertebral artery was not clearly visualized, and the right internal carotid artery showed a slightly lower signal intensity than the left internal carotid artery on the cervical and brain MRA. The course of the right brachiocephalic artery was not visible (Fig. 1A–B), which made us suspect the existence of aortic dissection in the time-of-flight MRA (TOF-MRA), but the definitive diagnosis could not be established (Fig. 1C). The subsequent pulse-gated noncontrast thoracic bSSFP-MRA revealed a dissection flap in the ascending aorta (Fig. 1D–E). Eventually, radiographs demonstrated mediastinal widening, and the chest CTA revealed Stanford TAAAD (Fig. 1F).

Emergency surgical aortic repair with the ascending aorta and arch replacement were performed. The patient was discharged in an ambulatory state without residual neurologic deficits.

## 3 Discussion

Contrast-enhanced CTA is the established modality for TAAAD diagnosis; however, CTA evaluation is not practical for all patients with an acute ischemic stroke or TIA, such as those with no chest pain or neurologic symptoms. Several markers, including systolic blood pressure laterality, elevated D-dimer, radiographic mediastinal widening, ultrasonographic common carotid artery dissection, and pericardial effusion, identify TAAAD in such patients [3]. Nevertheless, eliminating TAAAD based on a single finding is challenging because these markers do not have high sensitivity and specificity.

Recent advances led to contrast-free MRA techniques that rely on the blood flow to produce the desired vascular signal [4,5]. Among them, the 3-D bSSFP-MRA provides a rapid volumetric assessment and enables flow-compensated bright blood imaging with improved aortic visualization, facilitating dissection flap detection [4,6]. Thoracic MRA is more susceptible to artifacts, such as cardiac and respiratory motion, than other MRAs. Breath-holding helps overcome this drawback. TOF-MRA is limited by its long acquisition time and is unsuitable for aortic evaluation. Fig. 1D–E shows the better imaging quality of the pulse-gated noncontrast thoracic bSSFP-MRA compared with the chest CTA. Unlike CTA, noncontrast bSSFP-MRA can be routinely performed in cases of renal failure, iodine contrast media allergy, and pregnancy. Furthermore, it can simultaneously evaluate the brain and aorta. Additionally, the entire procedure takes less than 2 min, making it suitable for screening. Thoracic bSSFP-MRA might not be suitable for stroke patients with severe consciousness disturbances or Wernicke's aphasia, who cannot hold their breaths. In this respect, dynamic contrast enhanced MRA, which is usually performed within 40 s and does not require a breath-holding technique [7], is also one of the candidate screening sequences. However, it requires extra time to check the renal function before using the gadolinium-based contrast agents, and to date, there is no report comparing the usefulness of the noncontrast bSSFP and dynamic contrast enhanced MRA. Further studies are needed to validate the usefulness of each sequence.

## 4 Conclusion

Pulse-gated noncontrast thoracic bSSFP-MRA is one of a rapid and useful screening platform to rule out TAAAD when considering thrombolysis for acute ischemic stroke. However, further studies are needed to validate our findings.

## Grant support

This report received no specific grant from any funding agency in the public, commercial, or not-for-profit sector.

## Declaration of Competing Interest

None.
